# THE MEN IN DEMENTIA CAREGIVING: UNDERSTANDING MEN’S EXPERIENCES

**DOI:** 10.1093/geroni/igae098.0122

**Published:** 2024-12-31

**Authors:** Allison Lindauer, Kenneth Hepburn

**Affiliations:** Chair; Discussant

## Abstract

About 30% of caregivers for family members with dementia are men, and their numbers are rising with the increasing prevalence of dementia. However, there is limited information in the extant literature about the number of men providing care to family members with dementia, their experiences, and their engagement in caregiving research. What is known is concerning. Men, like women, experience burden in the caregiving role and are at higher risk for health problems than non-caregivers. In addition, men may experience role confusion and feel unprepared to assume the caregiving role. Finally, little is known about what motivates men to participate in dementia caregiving research. Here we describe men’s experiences from three groups: Black American men, men caring for family members with primary progressive aphasia, and men caring for family members with advanced dementia. Along with quantitative and qualitative data, we will present findings from focus groups which center on men’s motivations for joining research studies, perceived benefits of research participation, and men’s concerns about research, including biospecimen collection. Unique insights and needs from each group will be addressed, along with strategies to promote recruitment and retention of men in caregiving research.

